# Supplementary material for: Injection and sexual risk among people who use or inject drugs in Kampala, Uganda: An exploratory qualitative study
Source: PLoS One. 2020 Apr 22;15(4):e0231969. doi: 10.1371/journal.pone.0231969 (PMC7176107; doi:10.1371/journal.pone.0231969)
Supplement: S1 File — (DOCX) [file pone.0231969.s001.docx]

**Qualitative Interview with Drug Users**

Demographics

1. How old are you? __Years
2. Gender? Man__ Woman__ Transgendered__

Are you married? **Y N**

1. What is the highest level of school that you have completed?
   1. 0-3 years
   2. 4-6 years
   3. 7-9 years
   4. Some high school
   5. High school graduate
   6. College or higher
2. Which of the following best describes your current employment situation?
   1. Unemployed
   2. Employed part time in formal economy
   3. Employed full time in formal economy
   4. Employed part time in informal economy
   5. Employed full time in informal economy
   6. Housewife
   7. Retired
3. What is your current income? ____ week
4. **Interviewer READ**: Now I would like to ask you about various drugs that you use now or may have used in the past, the age you first used them, and how often you currently use them.

| Have you ever used _____? | How old were you the first time you tried ____? | How many *days* in the last 30 have you used ____? | How many *times* in the last 30 days have you used ___? | How many times did you use ___ *yesterday*? |
| --- | --- | --- | --- | --- |
| Alcohol yes/no |  |  |  |  |
| Marijuana yes/no |  |  |  |  |
| Glue/Paint yes/no |  |  |  |  |
| Crack yes/no |  |  |  |  |
| Sniffed Cocaine yes/no |  |  |  |  |
| Injected Cocaine yes/no |  |  |  |  |
| Methaqualone(Mandrax) yes/no |  |  |  |  |
| Sniffed Heroin yes/no |  |  |  |  |
| Injected Heroin yes/no |  |  |  |  |
| Methamphetamines yes/no |  |  |  |  |
| Tranquilizers(Xanax, Benzodiazepines) yes/no |  |  |  |  |
| Other yes/no  (Specify)  _______________ |  |  |  |  |

Introductory Questions

1. What is a typical day like for you? What do you normally do? How do you spend your time?
2. Has drug use affected your work in any way?

Drug Use Sites

1. Where did you use [drug or drugs mentioned] last? When?
   1. Tell me about this location. How frequently do you use this location? How did you come to know this location?
   2. Are there other people that use [drug or drugs mentioned] at this location when you do? Approximately how many people? Do the people in this location do other things along with using the [drug or drugs mentioned]? Do the people who use this location know each other? What do they do while they are using drugs (talking, fighting, having sex, etc.)? What do they talk about, why are they fighting, etc.?
   3. Besides [drug or drugs mentioned] what other drugs are used in this location?
   4. What do people have to do in order to use this location? Is there someone that controls the location? Are there rules to be able to use this location? What kind of rules? Do you have to pay a fee in money or drugs to enter this location?
2. Are there other locations that you go to use [drug or drugs mentioned] (abandoned buildings, homes, etc)?
3. For each of these additional locations (for each one mentioned), ask:
   1. How is this location and where is it located? **(No exact directions, but generally, is it inside or outside of the community)**
   2. How many times and how frequently do you use this location?
   3. Are there other people that use [drug or drugs mentioned] at this location when you do? Do the people who use this location know each other?
   4. Are there rules to be able to use this location? What are the rules?
   5. What other drugs do people use in this location?
4. Are there locations that you use more frequently than others to use [drug or drugs mentioned]? Which ones? Why do you go there more often?
5. In the last 6 months, have you started to use any new locations? **[If the response is yes]** Why?
6. Have you stopped going to locations that you used to go to use [drug or drugs mentioned]? **[If the response is yes]** Why?

Injecting

[If reports injecting drugs]

1. Tell me where you obtain needles or syringes to inject your drugs?
2. If you can’t get it at this location are there other places you go to get injection equipment?
3. How many times do you reuse your needles before you replace it?
4. Have you ever shared needles with anyone else? In what circumstances have you done this?
5. Have you ever measured drugs using a syringe which you then shared with someone else?
6. Describe how you prepare drugs for injection. Where do you get your water? What do you use to mix your drugs (spoon or bottle cap)? Does anyone else use the same thing (cooker) to mix their drugs?

Strategies for Obtaining Drugs

1. How do you [drug or drugs mentioned]? **[If purchased]** What do you do to get the money to pay for it?
2. When you cannot obtain [drug or drugs mentioned] this way, what other things have you done to get the drugs?
3. What type of jobs can users do for drug dealers?
4. Regarding these jobs, have you done any? Which? How were you paid?
5. Have you ever purchased [drug or drugs mentioned] for another person? How were you paid back?
6. Have you ever participated in the sale of [drug or drugs mentioned]? **[If participant has no experience selling drugs, move to Question 25]**
7. In what capacity did you participate?
8. In what type of locations did you sell [drug or drugs mentioned] or work for drug dealers? (**Expound:** street, buildings, etc)
9. Who did you sell drugs to? How did you decide who you would sell to? Did they have to be people you already knew? Did people from outside of the community come to purchase drugs?
10. Did you sell as part of a group? **[If the answer is yes]** Tell me about the group. Does it have a name? How big is it? Do members have different roles or assignments within the organization? What are they? What are the roles or assignments you had within the group? Was there a leader? Were there rules? What were the rules, and how were they enforced?
11. Have you ever exchanged sex for money, drugs, or something else valuable? What can you remember about the first time you have done so? And what do you remember from the last time?

Distribution System of Drugs

1. In what area of your community are drugs sold? Do you purchase [drug or drugs mentioned] in these locations? **[If the answer is yes]** What type of location is this? (On the street, in a house, etc). Why do you go to this location to purchase [drug or drugs mentioned]? **[If the answer is no]** Where do you buy drugs? What type of location is this? (On the street, in a house, etc). Why do you go to this location to purchase [drug or drugs mentioned] instead of a location in your community?
2. What other locations do you go to purchase [drug or drugs mentioned]?
3. What makes you choose where to go to purchase [drug or drugs mentioned]? (Price, security, credit, etc)
4. Do people from outside of your community come to purchase [drug or drugs mentioned]?

HIV and Drug Resources

1. Have you attempted to reduce or stop using [drug or drugs mentioned] at some point? **[If the response is yes]** How did you do it? Did you seek help? (From friends, institutions, others) Where did you go? Did you receive the help that you wanted?
2. Do you know of any organizations in Kampala or your community that help people who use [drug or drugs mentioned]?
3. Do you know anyone who has reduced or stopped using [drug or drugs mentioned]? How did they accomplish this?
4. Who are the people at greatest risk for contracting HIV? What do these people do to put themselves at risk?
5. Have you ever taken an HIV test? Where? How did you know about this place? Would you be comfortable sharing the results of your HIV test? Have you received information about how to prevent HIV? Who gave you this information? How? Have you been given condoms? Who gave you them?
6. Have you heard your friends or other drug users discuss HIV? What have they said?
7. Do you think that drug use is a risk factor for HIV? How?
