# Supplementary material for: Injection and sexual risk among people who use or inject drugs in Kampala, Uganda: An exploratory qualitative study
Source: PLoS One. 2020 Apr 22;15(4):e0231969. doi: 10.1371/journal.pone.0231969 (PMC7176107; doi:10.1371/journal.pone.0231969)
Supplement: Attachment — Submitted filename: PLosOne December 2 2019.docx [file pone.0231969.s002.docx]

Overall, this work helps fill an important gap in the prevention literature, which is severely lacking for this country. The work is presented cohesively. I do not have any major comments. Minor suggestions regarding syntax and grammar follow below.

Methods:

- Correct “people who used illicit drug use” in the first sentence
- Change “assess” to past tense
- Compound adjectives should be hyphenated. For example, “injection and drug related sexual risk” should be “injection- and drug-related sexual risk”.
- “HIV risky” should be “HIV risk”

Results

- The sentence “As a result, nearly all participants who injected drugs reported sharing injection equipment” is placed a bit prematurely in the paragraph—it only becomes clear why participants were reusing syringes in the following sentences. I would simply remove “As a result” to circumvent this issue. This also prevents repetition of this phrase within the paragraph.
- You may want to consider labelling quotes with “Interviewer” and “Participant ##” at the very least, to help the reader distinguish between speakers. Labelling by participant number also shows the reader that the quotes are not all derived from a small number of participants. There is some labelling throughout the section, but it is inconsistent.
- The paragraph that begins with “In this situation of scarcity” is a run-on sentence. Consider breaking this up into separate sentences to make it more digestible.
- The information about an external study conducted by the UHRN should not be presented in the results section. It can be linked to the results in the discussion section, as appropriate. Similarly, any discussion about the implications of the results, or framing context, should not be introduced in the results section.
- In the sentence which contains “reported returning syringes to users after injecting”, did the authors mean to say that syringes were returned to sellers after injecting?

Discussion/Conclusion

- There are some acronyms used in the conclusion which are not defined anywhere.
- Much of the conclusion seems to be discussion about implications of this research, rather than a conclusion proper. I would suggest restructuring this content so that the conclusion is more succinct, and the discussion includes the implications of this research and suggestions for future work.
