# Supplementary material for: Injection and sexual risk among people who use or inject drugs in Kampala, Uganda: An exploratory qualitative study
Source: PLoS One. 2020 Apr 22;15(4):e0231969. doi: 10.1371/journal.pone.0231969 (PMC7176107; doi:10.1371/journal.pone.0231969)
Supplement: Attachment — Submitted filename: PLOS revision letter.docx [file pone.0231969.s003.docx]

Dear Dr. Msafirir Francis and Reviewers,

Thank you for your careful review of our manuscript “Injection and sexual risk among people who use or inject drugs in Kampala, Uganda: A qualitative analysis.” The reviewers thought there was merit in the paper, but suggested a number of revisions which we outline below. Unfortunately, I did not read that we should submit a copy with track changes and one without, so I have highlighted the parts that were changed in the revised manuscript “with tracked changes.” We also address editor comments regarding format, data accessibility and making supplementary material such as interview guides available.

From Editor:

“When submitting your revision, we need you to address these additional requirements.
Please ensure that your manuscript meets PLOS ONE's style requirements, including those for file naming.” We have followed your format instructions in the revised manuscript.

“Please include additional information regarding the survey or questionnaire used in the study and ensure that you have provided sufficient details that others could replicate the analyses. For instance, if you developed a questionnaire as part of this study and it is not under a copyright more restrictive than CC-BY, please include a copy, in both the original language and English, as Supporting Information.” We did not use surveys but had brief demographic questionnaires at the beginning of the in-depth interview guide. We have included this and the interview guide in supplemental material as requested. This is available in English only. As an open-ended qualitative guide with flexibility in the order and wording of questions, we did not translate the guide into Luganda, but bilingual Luganda, English speakers translated the questions in the interview. Informed consent forms were translated to Luganda.

“Please provide additional details regarding participant consent. In the ethics statement in the Methods and online submission information, please ensure that you have specified how verbal consent was documented and witnessed.” We have included this information in the methods section and apologize for this oversight.

“We note that you have indicated that data from this study are available upon request. PLOS only allows data to be available upon request if there are legal or ethical restrictions on sharing data publicly. In your revised cover letter, please address the following prompts:
a) If there are ethical or legal restrictions on sharing a de-identified data set, please explain them in detail (e.g., data contain potentially identifying or sensitive patient information) and who has imposed them (e.g., an ethics committee). Please also provide contact information for a data access committee, ethics committee, or other institutional body to which data requests may be sent.
b) If there are no restrictions, please upload the minimal anonymized data set necessary to replicate your study findings as either Supporting Information files or to a stable, public repository and provide us with the relevant URLs, DOIs, or accession numbers. Please see [http://www.bmj.com/content/340/bmj.c181.long](https://urldefense.com/v3/__http:/www.bmj.com/content/340/bmj.c181.long__;!aSdVjyY7hSA!tQjtC-J4QIaEMOShrhD_VMH_9CeSm42neU-96H1Sq4yiydUfVetDNs2yiGgryrK2$) for guidelines on how to de-identify and prepare clinical data for publication. For a list of acceptable repositories, please see [http://journals.plos.org/plosone/s/data-availability#loc-recommended-repositories](https://urldefense.com/v3/__http:/journals.plos.org/plosone/s/data-availability*loc-recommended-repositories__;Iw!aSdVjyY7hSA!tQjtC-J4QIaEMOShrhD_VMH_9CeSm42neU-96H1Sq4yiydUfVetDNs2yiGqnTvUm$).” There are no legal or ethical restrictions to making data available except that it is often more difficult to remove all identifying information from in-depth interview data. We have begun depositing cleaned interview transcripts that will be made available through Qualitative Data Repository (QDR) at https://data.qdr.syr.edu/dataset.xhtml?persistentId=doi:10.5064/F6OM6HWD.

From Reviewer 1:

“While I recognize that this is a particularly difficult population to work with, I am not certain why recruitment could not have been more purposive in that only injection drug users were recruited. Instead, it is misleading to state that 30 participants were recruited, and then go on to describe only the experiences of the 17 among them who were actually injecting for some parts of the manuscript and more general experiences of the other users in other parts of the manuscript. Infact this demarcation is not at all clear. It is also unclear if saturation of themes were achieved with these 17 interviews (among injecting PWID), which the authors themselves admit is a limitation. This is a major methodological flaw in the study. Instead the authors should aspire to conduct a more robust qualitative study, recruiting enough injecting (male and female) PWID to explore these areas in detail until saturation of themes is achieved. Respondent driven sampling (which the authors used partially) can be a powerful way to recruit participants. The experiences of male and female PWID also need to be more clearly delineated since there is enough data to suggest that HIV disproportionately affects female PWID. Barriers to receive harm reduction services also differ significantly between male and female PWID. Instead what appears in this manuscript is the experiences of male and female PWID inter-mingled such that the sexual risk behaviors among female PWID (who are also sex workers) is explored in detail, and yet little is known about the sexual risk behaviors of male PWID. The other alternative is to focus exclusively on female PWID (as has been done in other studies in other settings) as a separate manuscript.” We believe that it was important, given the scarcity of knowledge about illicit drug use in Uganda, to include both PWID and PWUD but did not inject. Both groups engaged in sex exchange and other sexual-risk behaviors as we now clarify. The additional risk faced by women PWID and PWUD are in the frequency in which they exchanged sex, as many considered sex work as their main means of support. We believe that we received saturation of themes for injectors and non-injectors, women and men.

Reviewer 1 suggested several improvements in organization, including the following.

“The qualitative methodology needs to be explained in greater detail. Representative questions are presented (and can perhaps be organized under broad headings in a table); however not all questions are sufficiently dealt with in the results. For example, the authors state that they explored transitions to drug use and why injecting did or did not occur but do not describe this in the results.” We have taken out descriptions of questions which were not analyzed or presented in this paper. The entire interview guide is included in supplementary materials.

“Parent and child nodes for coding can be presented in a table.” We have included the coding tree in a Table in the manuscript.

“At the very beginning, in the introduction, a pre-amble can be provided on the following: i) size estimates of PWID in Uganda (at least what is known), ii) existence of syringe exchange programs and opioid agonist therapy services, iii) targeted intervention programs for PWID, iv) voluntary testing and counseling centers, iii) government ART centers. While this data is offered in the manuscript, it is interspersed in the results and discussion. Whereas describing this information at the beginning provides clarity and helps the reader recognize what is available. Subsequent themes on barriers to receive services (including paucity of services) or other factors can then be appreciated in this context. The authors note that a "package of interventions" is recommended for PWID -- indeed, this is also the WHO's recommendation for key populations (given the challenges in reaching and linking such hard to reach populations). Presenting what is available in Uganda, and noting that as is the case in many countries, service delivery to key populations often tends to be fragmented (even if services are available) will offer context as well.” The first paragraph of the paper included the size of the PWID population in Uganda, existence of syringe exchange programs and MAT, and targeted intervention programs for PWID. We have included information on government ART centers and HIV testing through UHRN. We have also described the developmental phase of these programs, and the lack of coordinated services in most LMICs.

“The quotes in the results section are interspersed with paragraphs such as data on clean water in Uganda. Firstly, for each theme that is explored, representative quotes can either be provided in text, or additional quotes can be provided in table format (or in an appendix). Secondly, the addition of explanatory data is distracting, and should only be reserved for the discussion section if relevant. The results section should be solely limited to the exploration of themes and presentation of quotes.” This was also a comment made by Reviewer 2. We have taken out all references to previously published research and implications of results to the Discussion section.

“There are numerous grammatical and spelling errors throughout the manuscript, and shifting from past tense to present tense within the same paragraph.” We have corrected these errors.

“The authors note that they recruited 30 participants but state that they conducted 31 interviews. This is perhaps a typographical error?” Indeed, this was a typographical error. We conducted 30 interviews and this is now consistent throughout.

“Examples of well written qualitative papers exploring similar issues among PWID in low and middle income countries can serve as a guide for how to write this manuscript (see below for some references). Overall it is an important area of study. With more robust recruitment and methodology, this submission can emerge as a much stronger and valuable manuscript in the literature:

Kermode M et al. Harm Reduct J. 2007 Dec 5;4:19. My first time: initiation into injecting drug use in Manipur and Nagaland, north-east India.

Kermode M et al. BMC Int Health Hum Rights. 2013 Jan 29;13:9. Falling through the cracks: a qualitative study of HIV risks among women who use drugs and alcohol in Northeast India.”

We thank the reviewer for this suggestion and carefully read the manuscripts.

From Reviewer 2:

“Methods:
• Correct “people who used illicit drug use” in the first sentence
• Change “assess” to past tense
• Compound adjectives should be hyphenated. For example, “injection and drug related sexual risk” should be “injection- and drug-related sexual risk”.
• “HIV risky” should be “HIV risk”

We have made all these changes.

Results
• The sentence “As a result, nearly all participants who injected drugs reported sharing injection equipment” is placed a bit prematurely in the paragraph—it only becomes clear why participants were reusing syringes in the following sentences. I would simply remove “As a result” to circumvent this issue. This also prevents repetition of this phrase within the paragraph.
We have followed this suggestion.

• You may want to consider labelling quotes with “Interviewer” and “Participant ##” at the very least, to help the reader distinguish between speakers. Labelling by participant number also shows the reader that the quotes are not all derived from a small number of participants. There is some labelling throughout the section, but it is inconsistent.

We have followed this suggestion and include Participant ID numbers to distinguish participants.

• The paragraph that begins with “In this situation of scarcity” is a run-on sentence. Consider breaking this up into separate sentences to make it more digestible.
We have broken up this sentence.

- The information about an external study conducted by the UHRN should not be presented in the results section. It can be linked to the results in the discussion section, as appropriate. Similarly, any discussion about the implications of the results, or framing context, should not be introduced in the results section

We have removed all instances of external studies referenced in the results section and moved them to the Discussion. This includes the information about water quality, the effectiveness of peer led interventions as well as the UHRN study mentioned.

• In the sentence which contains “reported returning syringes to users after injecting”, did the authors mean to say that syringes were returned to sellers after injecting?
Yes, we have corrected this mistake.

Discussion/Conclusion
• There are some acronyms used in the conclusion which are not defined anywhere.
• Much of the conclusion seems to be discussion about implications of this research, rather than a conclusion proper. I would suggest restructuring this content so that the conclusion is more succinct, and the discussion includes the implications of this research and suggestions for future work.

We have moved much of what was formerly in conclusions to the discussion section as the information in conclusions discussed implications of findings. We have also defined all acronyms. The conclusion now only contains a brief summary of findings.

We hope that these changes adequately address reviewers’ concerns.

Sincerley,

Julia Dickson-Gomez

Professor of Epidemiology

Institute for Health and Equity

Medical College of Wisconsin
