# Supplementary material for: Injection and sexual risk among people who use or inject drugs in Kampala, Uganda: An exploratory qualitative study
Source: PLoS One. 2020 Apr 22;15(4):e0231969. doi: 10.1371/journal.pone.0231969 (PMC7176107; doi:10.1371/journal.pone.0231969)
Supplement: Attachment — Submitted filename: PLOS ONE response to reviewers II.docx [file pone.0231969.s004.docx]

Dear Dr. Msafirir Francis and Reviewers,

Thank you for your careful review of the paper which was found to be improved but still in need of some clarification, particularly with regards to whether we achieved data saturation. We agree with Reviewer 1’s comments and do not claim to have reached data saturation and changed the title and limitations accordingly. We have also included access as well as availability of harm reduction materials where appropriate and corrected typos and grammatical errors.

*In this revision, the authors have been more transparent about their rationale to recruit both people who inject drugs and people who use drugs; the study also clearly includes both genders which is certainly valuable. In their response to my concern that the small number of participants may not have facilitated achievement of saturation of themes, the author state the following: “We believe that we received saturation of themes for injectors and non-injectors, women and men”. I want to point out that this directly contradicts what they write in the limitations section of this manuscript: “The present study has several limitations including the small sample size characteristic of qualitative studies. Given that not all participants injected drugs, we may not have reached saturation of themes regarding drug injection risks”. Also, I should note that nowhere in their methodology have they stated that they actually continued recruitment till saturation of themes was achieved.*

*I would suggest the following changes:
a. Small sample sizes are not a characteristic of qualitative studies. There are qualitative studies that include several hundred participants. It is best not to suggest this. What is true however is that, in general most investigators would halt recruitment when they start hearing the same themes (i.e., achieve saturation).
b. So instead of saying “we may not have achieved saturation of themes for certain topics”, and then stating (at least in their response ) that they did in fact achieve saturation, it’s best to perhaps characterize this as an exploratory qualitative study that despite its small numbers offers insights into risk behaviors among people in a neglected key population in need of services. The title of the manuscript can also be reworded as such: “Injection and sexual risk among people who use or inject drugs in Kampala, Uganda: an exploratory qualitative study”.*

We have made the suggested change in wording in the limitations section and the title of the paper.

*While the results section has been reorganized, I think this section can further be improved. Again, explanations for certain observations is not needed in this section. Please see below for suggestions on how to improve this section.. Throughout the manuscript, the term “availability” of harm reduction services is used. I think what the authors are actually assessing is whether PWID accessed harm reduction services. As they state in the introduction, there are few if any harm reduction services that are available. What is also true is that few PWID are actually accessing even the available services.*

We have included accessibility along with availability when appropriate. For example, HCV testing and Naloxone is currently not available. Clean syringes, HIV testing and condoms are available but not always accessible or accessed. We have tried to clarify these differences throughout.

*Acronyms are used throughout the manuscript. However, they should be described in full the very first time they are used. Please ensure this is the case. Also, after an acronym is already used, there are places where full forms are still presented – for example: Syringe exchange programs.*

We have spelled out all acronyms the first time they appear.


MINOR Comments:

*Abstract:
Background: Suggest modifying last statement as: “The objective of the research was to document injection and other drug related-risk behaviors among people who use drugs in Uganda to develop comprehensive HIV/HCV prevention intervention.*

We have edited the sentence as suggested.

*Methods: This section needs more details on qualitative methodology. Again, the term “availability” is used to describe both injecting equipment and harm reduction services. I believe the authors meant to say PWUD/PWID access of harm reduction and drug treatment services.*

We mean both availability and accessibility and have included both terms. As stated before, some services are currently not available like MAT, Naloxone, or HCV testing, others are available but so difficult to access as to make them virtually unavailable (e.g. the availability of clean syringes in pharmacies but their unaffordability, and the unavailability of SEPs except for the pilot study.

*Results: Should include the demographic characteristics at least briefly – how many were recruited etc. UHRN – acronym is used. Please provide full form following which acronym can be used in the rest of the manuscript.*

Demographics of the sample are included in a table. Acronyms are spelled out when first used.

*Conclusions: Comprehensive (?drug use) prevention and treatment services. “Linkage and retention to HIV care” should be reworded for grammar: “ Linkage to and retention in HIV care”.*

We have corrected this error

*Introduction:
transshipment points: Trans-shipment (with a hyphen)*

We have corrected this error

*PEPFAR COP2018 – While many are familiar with PEPFAR, what does COP stand for?*

COP stands for Country Operational Plan. We omitted this since it did not seem necessary for comprehension.

*“Most researchers argue that a combination of approaches are needed including: provision of clean syringes through syringe exchange programs (SEPs); frequent HIV testing and linkage to HIV care; antiretroviral therapy initiation after infection to reduce risk of onward transmission through viral suppression; medication assisted therapy; psychosocial support and pre- and post-exposure prophylaxis” : A colon is not needed after including and every point can be separated by commas.*

We have made this change.

*“harm reduction centers to the rest of the country and include MAT, syringe exchange and PrEP”: sentence should be rephrased as “ harm reduction centers to the rest of the country that include services such as MAT, syringe exchange and PrEP.*

We have made this change.

“*The current study presents results from qualitative interviews with 30 people in Kampala, Uganda who use injection and non-injection drugs to determine their behavioral risks for HIV and the availability of resources to reduce the harms associated with injection such as clean syringes, non-pharmaceutical drug treatment, condoms and HIV testing”. I am not sure what non-pharmaceutical drug treatment actually means? Buprenorphine for example is a pharmaceutical drug and is used in MAT programs.*

Non-pharmaceutical treatment includes Cognitive behavioral therapy, or even self-help groups and 12-step programs. We included these because MAT was not yet available in Uganda.

*“We include both people who inject drugs (PWID, n=17) and people who use drugs (PWUD) but do not inject (n=14), because while injection with contaminated syringes is one of the most efficient ways to become infected with HIV, PWID and PWUD can be at risk for becoming infected through highrisk sexual behaviors. In addition, PWID and PWUD often are parts of the same drug using networks”. This should be moved to methods.*

We moved this to the Methods Section.

*Eligibility criteria and interview sampling characteristics

“We purposively oversampled to include more women than in the general drug using population, and injection and non-injection users and from each community”. This sentence is not clear and am not sure what this is supposed to mean. With overall small sample, I am not sure this is accurate either.*

We meant that we recruited both injection and non-injection drug users and attempted to oversample women to get their perspectives. We did not attempt to sample from each community and we have corrected this misunderstanding.


*In-depth interview procedures and content:
“any experiences with harm reduction services such as clean needles and HIV testing”: I think the authors meant to say, “harm reduction services such as SEP and HIV testing”.*

You are correct and we have made this change.

*5. Qualitative analysis: The coding tree while helpful has some categories that clearly are based on the interview guide, and other terms that appear more random.*

We now highlight that the coding tree came directly from research questions as well as unexpected themes in the data such as Butabika, the mental hospital where some had received drug treatment, and which was widely feared by drug users. Similarly, we added drug confiscation as this was reported as drug users reported that police frequently confiscated drugs.

*6. Results:
This section should just start with the descriptive details of patient characteristics. Other sections go on to elaborate on the various risk behaviors – so a summary does not need to be provided here.*

We eliminated a description of the risk behaviors and only include the participant characteristics.

*Headings need to be broader and consistent with what you sought out to explore. For example, Syringe sharing, use of unclean water/ mixing practices will fall under a broader category of Injection related risk behaviors.*

We have put Syringe Sharing and Use of Unclean Water/Mixing Practices under Injection Risk. We relabeled Sex Exchange and Sex-Related Risk behaviors.

*This entire section is written in present tense and is not consistent with the tense used in the rest of the manuscript*

Most of the section was written in the past tense with some general statements such as “Naloxone is not currently available” in the present tense. We have corrected the few instances in which this was not the case.

*There are some typographical errors: “Some PWID sterilized their water for injecting by boiling it themselves, getting in pre-boiled from their dealers, or purchasing sterile water from pharmacies”. I think the authors meant to say “getting it pre-boiled”.*

We believe we have caught and corrected the typographical errors.

*Again, please avoid providing explanations or consequences in the results section. For example: “If drugs, syringes, or spoons are shared after mixing drugs with blood, the risk for contracting HIV/HCV is high”.*

We have eliminated this.

*Harm reduction services: There is suddenly a mention of an individual by name of “Ronald”—I am presuming that this was actually participant 7 who is identified as a peer syringe distributer and then is mentioned as being Ronald. It may suffice to just state that those who were peer educators experienced benefits without having to name Ronald.*

Ronald, as mentioned in the original manuscript, was a pseudonym. However, we have changed the quotes to say [UHRN peer educator]. I do not know if Participant 7 and “Ronald” are the same person as we did not collect personal information like names.

*Overdose prevention: “Workers at the UHRN and participants confirmed high rates of overdose from heroin use”. Theoretically, workers at UHRN were not part of this study, unless they also participated in interviews. It may be better to present overdose data collected by UHRN in the discussion if available and stick to just participant descriptions in this section*

We have made this change.

*Quitting or reducing drug use: “In part this may because people who use drugs have not been given information about drug treatment, either through MAT, or by coping with withdrawal and cravings in the case of cocaine addiction”. This sentence is grammatically incorrect.*

We have corrected this sentence.

*7. Discussion:

“While harm reduction efforts have mainly focused on PWID in the developing world, injecting with contaminated water may increase the risk of abscesses, sepsis, endocarditis or other infections associated with injection” – This is a repetition (already mentioned earlier in the paragraph).*

We have eliminated this redundancy.

Reviewer #2: Dear Author,

This manuscript presents a significant contribution to the literature around injection-related risk behaviors for HIV and other blood borne pathogens among PWID and PWUD in Uganda. As a formative study, this qualitative work paints a broad picture of the context of drug use among some PWID and PWUD in Uganda, and clearly delineates avenues that should be pursued in further work. The authors addressed all of my comments. I recommend that the manuscript be accepted.

*A small note--during proofing, in the first sentence of the conclusion, change 'in' to 'is'.*

We have corrected this typo.

We hope that the revised manuscript has addressed all remaining concerns.

Sincerely,

Julia Dickson-Gomez
