# Supplementary material for: Injection and sexual risk among people who use or inject drugs in Kampala, Uganda: An exploratory qualitative study
Source: PLoS One. 2020 Apr 22;15(4):e0231969. doi: 10.1371/journal.pone.0231969 (PMC7176107; doi:10.1371/journal.pone.0231969)
Supplement: Attachment — Submitted filename: Plos_one review.docx [file pone.0231969.s005.docx]

I only have minor comments for the authors. Would recommend that the journal proof-read for grammar and typographical errors.

Abstract:

“ Availability and accessibility of syringes” – amended to “ Availability and accessibility of clean needles and syringes”

Introduction:

“While Uganda has been the site of abundant prevention and research efforts” – include the term “ HIV prevention and research efforts”.

“it is one of only two countries in Sub-Saharan Africa (SSA) in which incidence is increasing” – include the term “ HIV incidence”.

“In Kenya, of 101 current injectors, 52.5% were found to be HIV positive, attributed mainly to sharing injection equipment compared to 13.5% of heroin users who had not injected” – include reference

“In 2018, the Kampala Place Study estimated over 18,000 people who inject drugs (PWID) in Uganda” – include reference

“12-step prgrams as MAT was not available at the time of study” – typographical error in programs. “As MAT was not available at the time of study “ should be in parentheses

Methods:

“Some codes, such as Butabika and drug confiscation by police authorities emerged from the interviews and were added after careful reading of transcripts”. This can be re-written to just say that new themes that emerged were included in the final coding scheme.

Results:

Injection risk behaviors:

“In this situation of scarcity, even people who know the risks of sharing and take precautions to protect themselves reported that they would share their used syringes with other users” – modify tense: In this situation of scarcity, even people who knew the risks of sharing and took precautions to protect themselves reported that they would share their used syringes with other users.

“Syringes were also be shared when PWID bought premeasured syringes from drug dealers” – grammatical error. Should be modified as “Syringes were also shared when PWID bought premeasured syringes from drug dealers”.

“Unfortunately, the pilot lasted only a year and funding for it had just ended by the time the interviews for this project were conducted, and many participants reported that they had not seen Ronald or received syringes from him for some time”. Ronald is suddenly introduced here. I think this can just be limited to saying, “Unfortunately, the pilot lasted only a year and funding for it had just ended by the time the interviews for this project were conducted”.

“Some have heard that HIV is no longer a problem because everyone already has it and there is effective medication”. Change to past tense for consistency with rest of paragraph: Some had heard that HIV was no longer a problem because everyone already had it and there was effective medication.

“The woman reported that she “can’t” go because she is too afraid and will only go if she is confronted by symptoms”. Again, change to past tense
