# Supplementary material for: Injection and sexual risk among people who use or inject drugs in Kampala, Uganda: An exploratory qualitative study
Source: PLoS One. 2020 Apr 22;15(4):e0231969. doi: 10.1371/journal.pone.0231969 (PMC7176107; doi:10.1371/journal.pone.0231969)
Supplement: Attachment — Submitted filename: Uganda response to reviewer comments.docx [file pone.0231969.s006.docx]

We thank the reviewers for their thoughtful and encouraging response to our proposed research “Integrating buprenorphine into a multi-component HIV prevention intervention for people who inject drugs in Kampala, Uganda.” The reviewers felt that it was highly significant and innovative as one of the first multi-component HIV prevention interventions in Sub-Saharan Africa with considerable potential to reduce HIV/HCV incidence. They thought the approach was strong, particularly the use of the Consolidated Framework for Implementation Research used to adapt the intervention into the organizational context of two Drop in Centers already providing services to PWID in Kampala. The reviewers’ raised some concerns which we address below.

**Reviewers felt that not enough attention was paid to structural factors and gender dynamics that might affect the accessibility of the intervention.** The first phase of the project includes adapting the combination intervention to be feasible in the DIC contexts. We deliberately chose two DICs that differ according to overall resources and one that exclusively serves WWID, particularly female sex workers. CAB meetings will include members of each of the organizations. Discussions with the CAB will focus on the five domains of the CFIR including the intervention characteristics, inner setting, outer setting, characteristics of the population and implementation strategies. All these domains may include structural barriers and facilitators and gender dynamics, and this process will allow us to address these in the most appropriate ways. For example, an examination of the implementation strategies may indicate that transportation to the DICs is a major obstacle for participants to receive an HIV/HCV test and thus receive other services. Similarly, CAB meetings will focus on the particular needs of WWID, including those who engage in sex work, and could include the need for additional services such as child care while at the clinic, places to shower and for personal hygiene (already provided at AWAC) and resources and strategies to avoid gender based violence.

**Reviewers also suggested we include PWID and WWID to help address structural factors, gender dynamics and HIV and drug-related stigmas that might affect the accessibility of the intervention.** In our proposal, we stated that members of the CAB would include peer educators but did not make clear that they are PWID. Their roles as peer educators and PWID will allow them access to a number of opinions from PWID in their communities and barriers and facilitators to services through their direct experiences in providing them. The CAB will also include PWID who are not peer educators to include perspectives of harder to reach PWID who may not be engaged in services at the DICs (see Reviewer 2 comment about people with lived experience).

In addition, In Phase 1 the proposed 6 focus group interviews with PWID who do and do not use services at the DICs will cover proposed intervention components and anticipated structural barriers (e.g. transportation, childcare, stigma) that might prevent them from participating in the intervention.

**Reviewers felt that secondary distribution of buprenorphine (diversion) was not sufficiently explored nor were plans to deal with this described.** We will use a combination buprenorphine, naloxone medication to reduce the potential for abuse, overdose and diversion. Because of the addition of naloxone, combination buprenorphine/naloxone medications have a very low potential for overdose, even in combination with benzodiazepines (which we did not find as a drug of choice in our qualitative work) or alcohol use. Most combination medications like Suboxone, when diverted, are used by drug users to avoid withdrawal symptoms if they cannot obtain heroin, or to cut down or reduce their heroin use. Additional measures we will use include the following: Screening and addressing concurrent substance use disorders; structured intake process and treatment agreement screening for and discussing all potential harms and benefits of treatment; particular focus on the risks/benefits of initiating buprenorphine for those using other sedatives and counseling to avoid concurrent use; regular urine drug screening including for buprenorphine; safety procedures (safe storage plan, secure prescribing plan). If diversion is definitively proven, treatment agreement would state this could be grounds for treatment team (at their discretion) to discharge patient from treatment. We will also collect information from PWID in the CAB and in focus group discussions to discuss how buprenorphine might be diverted and what should or could be done to address this.

**The reviewers had many questions regarding the use of RDS in the HIV/HCV testing including whether RDS was appropriate in Uganda, as we used our experience in El Salvador, and would result in sufficiently long chains of recruitment.** The CRANE surveillance surveys conducted by our Investigative team in Uganda have successfully used RDS to recruit female sex workers (FSWs) and other key populations. They recruited 1487 FSW between April and December of 2012, starting with 4 seeds who were selected because they were well known and respected among sex workers. They had a maximum recruitment chain length of 25 waves. FSW and female PWID have considerable overlap, so we expect that we would be similarly successful in Uganda (Doshi et al., 2018). In addition, our qualitative research with PWID suggests personal drug use networks of between 15 to 20 people, larger than those we found in El Salvador. We therefore expect to have little difficulty in recruiting using RDS but will adjust methods if needed. However, it should be noted that the assumptions regarding RDS only need to be met to estimate HIV/HCV rates in the *population of PWID*, which is a secondary aim. If RDS chains are not sufficiently long, we will use GEE to adjust for the recruitment method and estimate HIV/HCV prevalence for participants in the study only. **Related to this, reviewers asked for more detail regarding how seeds would be selected, whom they would recruit, how long the referral chains would be and if there would be a limit.** We will select seeds to represent different drug “hot spots” that are located in informal settlements in Kampala. We will purposively recruit seeds for diversity on age, gender and participation in active sex work and select those who are well known and respected in their networks. Previous studies have identified 81 hotspots in Kampala. We will randomly select 6 and select 2 to 3 seeds from each. We will reseed in additional communities if recruitment waves end. We are limited by time, not number of participants and so will reduce the number of coupons given during the last month of the intervention, or sooner, depending on the rate of HIV/HCV testing. **Reviewer 3 asked how participants would select network members to receive an HIV/HCV test.** Participants will complete a short survey of their injection drug use network members, i.e. people with whom they use drugs, and will be asked to recruit from this list. However, those who are recruited if they are PWID will also be given an opportunity and coupons to recruit PWID for an HIV/HCV test. Thus, the HIV/HCV testing intervention resembles a referral chain such as RDS rather than an ego network. If this recruitment strategy does not work in producing multiple waves of recruitment, alternatives are presented in the proposal such as venue-based or mobile van testing at drug use hotspots. Recruitment for this project occurs through the HIV/HCV social network intervention. **Reviewer 3 asked what data we would use to avoid duplication in RDS chains.** We will collect locator information including name, age, gender and any personally identifying features such as scars or tattoos. This will not guarantee eliminating duplicate entries, but allowed us to find and remove over 200 duplicate participants in our research in El Salvador. **Reviewers also questioned how the social network data collected in Phase 2 would be used.** Social network data will be used to track who recruited whom and the size of participants’ networks in order to determine the probability that participants were selected to estimate HIV/HCV prevalence. This will allow us to estimate the prevalence of HIV/HCV among PWID, if RDS assumptions hold.

**Reviewer 1 questioned the use of monthly HIV tests as an intervention outcome.** Monthly HIV/HCV tests serve as the best measure of an increase in reach to PWID during the intervention period. This is because UHRN has provided HIV testing for many years and, thus, HIV monthly testing rates can be used as a baseline to evaluate increases in reaching PWID after intervention implementation. We will also do this for syringe exchange, but UHRN has done this for a shorter period and at a much smaller scale, and so syringe distribution will not give us as much information about increases in intervention reach. HIV/HCV testing is a way of not only providing an important service but is also a way of linking PWID with and without HIV to appropriate services to reduce the spread of HIV.

**Reviewer 1 questioned the use of summative risk behavior scores, instead of looking at individual risk behaviors which may differ based on participation in different components.** We agree and will look at individual and summative sexual risk and injection risk scores in outcome analyses.

**Reviewers also felt that polydrug use needed to be better addressed in the intervention and analysis.** We will offer CBT4CBT to any participant who wishes to stop using drugs including opioids, marihuana and other drugs. We will also include polydrug use in analysis.

**Reviewer 2 felt that the peer navigation part of the intervention was poorly described.** Peer navigation is already available at UHRN and will be made available at AWAC. Peer navigators accompany newly diagnosed PLH to their health care appointments and then follow-up with them to ensure that they are maintained in medical care. Our research in Uganda with PLH and HIV medical providers showed a strong relationship between UHRN and four HIV clinics, who also contact UHRN peer navigators to help find PLH who were tested at UHRN and who have dropped out of care.

**Reviewer 2 felt that more overdose prevention in addition to Naloxone was needed in the intervention. Similarly, Reviewer 1 suggested that more emphasis be placed on other health problems caused by injection drug use such as skin and soft tissue infections and endocarditis.** We will include a comprehensive overdose prevention training including tips such as never injecting alone, taking a smaller dose before injecting an entire dose, and recognizing symptoms of overdose and how to administer Naloxone. Naloxone will be offered to all PWID who inject heroin. Dr. Bryan Johnston is a physician with considerable experience working with PWID, and UHRN and AWAC will have full time medical personnel on site. They will be trained on lancing abscesses, prescribing antibiotics for skin or soft tissue infections and recognizing the symptoms of bacteremia and endocarditis and when to refer patients to hospital.

**Reviewer 2 felt that the roles of the Key Personnel need to be better described.** Dr. Dickson-Gomez will provide overall research, training, and technical leadership and direction to the project; participate on all technical committees of this project; supervise fiscal management of this grant; guide collection, management, and analysis of project data; assure compliance with the MCW, MaSPH and Uganda IRBs; and prepare and publish scientific papers and project reports. Dr. Glasman will serve as co-Investigator in this project and will help develop the protocol for the HIV/HCV social network testing intervention, help integrate other components into the combination prevention, analyze quantitative and qualitative data and publish scientific papers and project reports. Dr. Sergey Tarima will serve as the lead biostatistician on the project and direct and oversee all quantitative analyses. Dr. Johnston is a family medicine physician who has experience in training primary care physicians to prescribe buprenorphine to patients with opioid use disorder. He also has clinical and research experience in Uganda. His role will be to provide training to physicians to provide buprenorphine, setting up protocols to avoid buprenorphine diversion, and train medical personnel in the DICs to manage skin and soft tissue infections and other complications such as endocarditis. Dr. Musinguzi will serve as the Uganda PI. As such he will be responsible for overseeing data collection and intervention protocols in the UHRN and AWAC harm reduction drop in centers, data management and quality control, and will participate in data analysis, publication and presentation of study findings in scientific journals and meetings. Moses Ogwal, will serve as a project coordinator. He is a surveillance officer at Makerere University School of Public Health – Crane Survey project with vast experience in Key populations programming. Mr. Ogwal will be responsible for communicating with field researchers at the UHRN and AWAC drop-in centers, overseeing data quality and intervention fidelity, and producing progress reports. Wamala Twaibu is the director of the Uganda Harm Reduction Network (UHRN) and will serve as a co-Investigator on the project. He will be responsible for facilitating the CAB groups, the CFIR process and running PDSA cycles at the UHRN and AWAC drop-in centers, with the help of other co-Investigators. In addition to these Key Personnel we have two consultants. Kathleen Carroll is the developer of CBT4CBT, the computer program that will be used to support drug users who wish to stop using drugs in this intervention. She will help in the cultural adaptation and translation of the intervention. Rhoda Wenyenze, Dean of the School of Public Health, Makerere University, will also serve as a consultant on the project. She has served on various technical committees of the Ministry of Health and other agencies in Uganda including the Ugandan Ministry of Health (MoH) HIV counselling and Testing Research and Policy committee, the HIV Strategic Information and Key Populations committees. She has also served on Boards of four large HIV non-Governmental organizations in Uganda and is currently a Board member of the Uganda AIDS Commission. In her role as consultant, she will leverage these linkages with the MoH, development partners, and other HIV agencies within Uganda to provide an opportunity for wide stakeholder engagement which is critical to evidence uptake to contribute to HIV epidemic control in Uganda.

**Similarly, s/he asked how much the Uganda-based co-Investigators would contribute to and be involved in the protocol development and CFIR process.** All Uganda investigators will be intimately involved in the CAB groups, sessions analyzing the PDSA cycles and discussions to improve the fit and implementation of the intervention. Wamala Twaibu, as the director of the UHRN DIC and as an advocate to expand health services to drug users will facilitate discussions with staff at the DIC. Dr. Musinguzi, Ogwal and Dickson-Gomez will prepare the results of the CFIR process and PDSA cycles for CFIR and PDSA discussions to provide data used to change the intervention.

**Reviewers were not clear on how qualitative interviews conducted in Phase 2 would be analyzed or how it would be used to change the intervention, if at all.** In our previous research, we have found it extremely helpful to interview participants after they have completed an intervention to gain further insight into components that worked or did not, and reasons why. For example, qualitative interviews with participants in peer led interventions for crack users in Baltimore and Hartford, CT revealed that outreach to some participants was dangerous and inappropriate (Julia Dickson-Gomez, Knowlton, & Latkin, 2003), such as younger drug sellers, and that participating as a peer educator was seen as a job and led peer educators to reduce their drug use (Julia Dickson-Gomez, Convey, & Weeks, 2011; Julia Dickson-Gomez, Knowlton, & Latkin, 2004). We expect that these insights will help us to further refine the intervention for a subsequent R01. **Similarly, how implementation outcomes would be analyzed to revise the intervention was not described.** We consider implementation outcomes to be essential in testing the feasibility and acceptability of the pilot, and in planning for a full-scale trial and scale-up in subsequent interventions. Implementation outcomes such as reach of the intervention, fidelity of implementation, uptake by the population will inform us whether additional resources are needed to address the need, and additional intervention adaptation is needed. For example, if reach of the HIV/HCV social network testing is good, but acceptance of buprenorphine is poor, we may add additional training in motivational interviewing, or social media campaigns of former PWID who have been successfully maintained on buprenorphine.

**Reviewer 2 found the lack of Ns in the intervention pilot distracting.** The social network HIV/HCV testing intervention is designed to increase the numbers of PWID who are reached by the DICs and offered intervention services. Thus, an increase in the numbers of PWIDs served is a major outcome of the research and it is difficult to predict a priori how well this approach will work in Uganda. We are optimistic given our previous experiences with RDS in El Salvador and Uganda that it will work well. However, we have developed alternative strategies if we cannot increase the number of PWID seen at the DIC by this method. PWID who enter the DIC for an HIV/HCV test will be offered an array of harm reduction services based on their needs and preferences. The upper limit of participants in each of these is determined by the PWID who come to the DICs, but likewise, the rate of acceptance cannot be determined a priori. It is for this reason that implementation outcomes are a major focus on the pilot study.

**There was a difference of opinion regarding the use of Electronic Medical Records with one reviewer considering it a strength and the other feeling it was an unnecessary invasion given the amount of self-report data we are collecting. Reviewer 3 raised this as a human subjects concern as well as the need for additional protections to safeguard confidentiality, like identification numbers rather than names on all materials that have health data.** Electronic Medical Records will be used only in aggregate and we will not link participants in the intervention to prior EMR. The purpose of the EMRs is to establish an increase in services provided after implementation of the multi-component HIV prevention intervention in the two sites. We hypothesize that more people will come to the DICs for HIV/HCV testing after social network testing and will then receive additional services such as clean syringes as a result. Unlike in the original proposal, we will not attempt to triangulate self-report data with EMRs. Rather, no personally identifying information will be taken from EMRs. In addition, we will use only identification numbers on all surveys and other project materials to further safeguard participant confidentiality.

**Reviewer 2 thought it was odd that the questions in the approach did not match aims.** The five questions in the description of Phase 2 of the approach are to answer Aim 3, “To assess the feasibility, acceptability and initial promise of the intervention.” Question 1 asks whether the number of participants reached with HIV/HCV testing and syringes increased after the intervention (feasibility and reach); question 2 asks whether appropriate intervention components (e.g. syringes for those who inject, naloxone for heroin users) were offered to participants (fidelity); question 3 asks about the rate of acceptance of intervention components (acceptability); question 4 asks whether injection and sexual risk behaviors decrease over time (initial promise of the intervention). Finally, question 5 is exploratory and will be used to estimate the HIV/HCV prevalence in the population of PWID (if RDS assumptions hold) or within participants.

**Finally, Reviewer 2 found the timeline confusing, particularly when 3-month follow-up interviews will occur.** 3-month follow-up interviews will occur 3 months after the first participant is enrolled in the project during the pilot of the intervention.

Dickson-Gomez, J., Convey, M., & Weeks, M. (2011). Social psychological processes of enhanced HIV risk reduction among peer interventionists. *Journal of Community Psychology, 39*(4), 369-389.

Dickson-Gomez, J., Knowlton, A., & Latkin, C. (2003). Hoppers and Oldheads: qualitative evaluation of a volunteer AIDS outreach intervention. *AIDS and Behavior, 7*(3), 303-315.

Dickson-Gomez, J., Knowlton, A., & Latkin, C. (2004). Values and Identity: The meaning of work for injection drug users involved in volunteer HIV prevention outreach. *Substance Use and Misuse, 39*(8), 1259-1287.

Doshi, R. K., Sande, E., Ogwal, M., Kuyingi, M., McIntyre, A., Kusiima, J., . . . Hladik, W. (2018). Progress toward UNAIDS 90-90-90 targets: A respondent-driven survey among female sex workers in Kampala, Uganda. *PLoS One, 13*(e0201352).
